# A Multichannel Convolutional Neural Network Architecture for the Detection of the State of Mind Using Physiological Signals from Wearable Devices

**DOI:** 10.1155/2019/5397814

**Published:** 2019-10-03

**Authors:** Sabyasachi Chakraborty, Satyabrata Aich, Moon-il Joo, Mangal Sain, Hee-Cheol Kim

**Affiliations:** ^1^Department of Computer Engineering, Inje University, Gimhae, Republic of Korea; ^2^Institute of Digital Anti-Aging Healthcare, Inje University, Gimhae, Republic of Korea; ^3^u-HARC, Inje University, Gimhae, Republic of Korea; ^4^Division of Computer Engineering, Dongseo University, Busan, Republic of Korea

## Abstract

Detection of the state of mind has increasingly grown into a much favored study in recent years. After the advent of smart wearables in the market, each individual now expects to be delivered with state-of-the-art reports about his body. The most dominant wearables in the market often focus on general metrics such as the number of steps, distance walked, heart rate, oximetry, sleep quality, and sleep stage. But, for accurately identifying the well-being of an individual, another important metric needs to be analyzed, which is the state of mind. The state of mind is a metric of an individual that boils down to the activity of all other related metrics. But, the detection of the state of mind has formed a huge challenge for the researchers as a single biosignal cannot propose a particular decision threshold for detection. Therefore, in this work, multiple biosignals from different parts of the body are used to determine the state of mind of an individual. The biosignals, blood volume pulse (BVP), and accelerometer are intercepted from a wrist-worn wearable, and electrocardiography (ECG), electromyography (EMG), and respiration are intercepted from a chest-worn pod. For the classification of the biosignals to the multiple state-of-mind categories, a multichannel convolutional neural network architecture was developed. The overall model performed pretty well and pursued some encouraging results by demonstrating an average recall and precision of 97.238% and 97.652% across all the classes, respectively.

## 1. Introduction

Biosignals or physiological signals are those signals that can provide the details about the physiological states and their associated dynamics in the body of a human being [1]. The biosignals can be further analyzed to detect the physiological state based on the time series analysis of the signal [2]. Till date, many researchers have highlighted the relationship between biosignals and its associations in several contexts such as emotional behavior, social behavior, and expressive behavior [3, 4]. Emotional feeling or emotional judgment, which is also a subsection of the state of mind, mostly gets enhanced due to the physiological responses and can be detected by analyzing explicit patterns of the biosignals [5, 6]. The emotional changes are found to be related with the biosignals such as the skin conductance and heart rate, and this relationship helps to interpret the states of emotion such as stress and the other states of mind [7, 8]. Therefore, the detection of the proper state of the mind for maintaining a balance with health is necessary.

In the past, the acquisition process of the biosignals was a very cumbersome process that primarily included a clinical environment with a huge number of sensors and moreover, the process was quite expensive altogether. But, after the advent of wearable technologies or smart wearables, which has grown into much popularity, it is now quite easier to fetch the data and analyze it [9]. Wearable devices also help to quantify the parameters in space and time that help to monitor the desired state depending on the application and the purpose.

In the proposed study, different physiological signals of the subjects are coupled together to detect each state of the mind more accurately and precisely. The complete study was performed by engineering state-of-the-art features and followed by applying a multichannel convolutional neural network for the prediction of the states of the mind. The major novelty of the work can be put forward in multiple ways. First, the data that have been used in the study have been fetched from multiple subjects over a long period of time [10]. Second, the data have been fetched by using two different devices, namely, a wrist-worn wearable and a chest-worn wearable device. The usage of two different devices allows us to fetch much more localized information from the data. Third, the engineering of the features has been performed using a peak detection technique, which allows us to understand cumulative information about the data for a particular cycle. And finally, for the learning task, a multichannel convolutional neural network was developed. The network allows different biosignals to pass through different channels for optimum feature learning and, at the end, provides the prediction probabilities by concatenating the feature maps of all the channels.

The rest of the paper is structured as follows: The second section provides the details about the related work that has been performed in a particular segment of stress detection, wearable technology, machine learning, and deep learning. The third section presents a deep understanding of the data preprocessing and feature engineering. The fourth section discusses the development of the deep learning model and discusses the training procedure. The fifth section provides the results that were achieved in the work followed by the sixth section, which discusses the complete inflow of data to the prediction and also explains the societal impact of the work. Lastly, the paper is concluded in the seventh section.

## 2. Related Work

In the past, many researchers have highlighted the importance of biosignals for detecting different positive or negative emotions and different mental states depending on the situations. In [10], a data set was introduced for the wearable and stress detection known as WESAD, which holds the very basic need for the work. The data set was obtained from 15 subjects who had undergone an experimental process of answering a set of question that analyzed the affective state of mind of a subject. The biological signal data from the subjects were extracted using two wearable devices, that is, a wrist-worn wearable device and a chest-worn pod. The complete experimental procedure managed to obtain the data for 5 different activities namely, baseline condition, amusement condition, stress condition, meditation, and recovery. The study also performed a machine-learning classification task and a comparative analysis between the multiple algorithms that have been considered. Furthermore, the complete classification task was divided into two categories namely, a three-class classification based on the baseline, stress, and amusement class, and a two-class classification based on stress and nonstress, respectively.

But, in a perceptive case, it can be widely assumed that the decision thresholds for identifying a particular state of mind may not be the same across all the times for a particular individual. Therefore, a normalization factor was devised in [11] to reduce the stress variability, which was primarily maintained to check the tradeoff between the physiological data and the biosignals of the individual. In the study, the author collected the data from around 10 subjects but in a different way, that is, the data were captured for 5 days using three devices namely, wristband, smart necklace, and a chest band. The manner in which the particular work is different from the work performed by others in stress detection is that the data extraction process is not performed in a controlled way or in a laboratory environment rather was extracted for the complete time of 24 hours and that too for 5 days. Therefore, it was suggested in the work that there are many physiological thresholds that differ from individual to individual and must be considered for determining stress in a person. Also, the author demonstrated the usage of few regression models to predict the amount of stress in a person, which also presented some astounding results.

Moreover, the type of activities that are pursued by the people also has a different perspective towards maintaining the decision threshold. While pursuing some strenuous activities such as driving, the amount of mental stress threshold for a particular person increases drastically from the normal state to the mobile state. Therefore, to answer this particular subjective scenario, a stress detection model was developed in [12] for drivers in the real world. In the study, a real-world driving task across 24 drivers for around 50 minutes each was performed. However, the complete study was based on the amount of stress undertaken by the driver on the route driven rather than the natural stress elements. Also, in [13], a novel method for the detection of psychological arousal while driving a car using smart wearable sensors was proposed. The work performed the data extraction process on 11 participants who underwent a driving simulation while wearing a wrist wearable device that propelled out the physiological signals such as heart rate, skin conductance, and skin temperature. These physiological signals were further trained on a convolutional neural network that outperformed other baseline neural network models and denoising autoencoder models.

Now, as discussed previously, a wide range of stress detection or stress classification has been performed for the driving activity but the relevance of the sensors used for deriving a particular outcome is one of the concerns. Therefore, [14] performed a study for the selection of features and also the sensors for the detection of stress in drivers. For the model development, supervised machine-learning classifiers were used for two different calibrations namely, the driver at rest versus driving and driving on the highway versus driving in the city. From the work, it was obtained that for differentiating between rest and driving, heart rate, EDA (electrodermal activity), and respiration came out to be the best sensors. And, for distinguishing between low stress and high stress, heart rate and respiration turned out to be the most relevant ones. However, the study also demonstrated an interesting method for plotting the interindividuality between the subjects by normalizing each feature using the standard deviation of all the features across all the patients.

The development of a generic model for the state of mind detection of different individuals seemed to be quite important as each individual has a wide range of different grant roots or thresholds for a specific condition. Therefore, for solving this particular scenario, a study was performed by [15] on the stress detection using the heart rate variability. The method produced a normalized approach to account for the interindividual physiological difference using a baseline methodology. In another scenario, a classification model was devised by [16] for the detection of presurgery stress in patients, as surgery is a strenuous situation and the adverse effects of stress on patients undergoing surgery are irrefutable. The study leveraged the electrodermal activity of the patients that were extracted using a wrist wearable. The data were fetched from 41 patients who underwent different surgeries. The model developed in the work was based on adaptive partitioning of the data for stress detection where the interindividual variability of the electrodermal activity of a person was based on the sweat gland density and skin thickness.

For the stress detection of individual using electroencephalography (EEG), [17] published a DEAP data for emotion analysis using EEG and physiological signals. The data were generated while the subject viewed a 40-minute clip of a video. And after the data fetch was completed, a learning process was initiated, which resulted in classifying the EEG signals into different emotional classes. Reference [18] proposed and demonstrated a method for determining the stress level for the patients suffering from dementia. The study collected data from a single wearable sensor attached to the subjects' body to classify the stress level in 6 classes. The study was performed on 36 subjects, of which 30 were normal people and 6 were patients suffering from dementia. The study was further validated with the observational data of the behavioral patterns that were extracted by the clinical staff and were matched with the threshold-based sensor data.

Furthermore, in the study regarding emotional stress detection using EEG signals, [19] leveraged deep learning algorithms to analyze the fluctuations of electrical activity in the brain. The data used in the study were captured from the test subjects using a NeuroSky device, while the test subjects were listening to the music. Furthermore, into the learning process, a backpropagation deep neural network was implemented for stress detection, which resulted in the accuracy of 80%.

As primarily, the studies performed for the detection of stress predominantly used the wearable devices and noninvasive sensors for the extraction of signals, therefore [20] developed a system for determining stress detection using the bioradar respiratory signals. The work implemented two unique approaches, one for the acquisition of signals and other for the engineering of the features. For the data acquisition process, a noninvasive and a noncontact method were devised, and for the engineering of the features, recurrence quantification analysis was performed. For the learning process, a multilayer perceptron was designed to perform a binary classification over steady and stress class, respectively.

The above-highlighted work further motivated us to explore the proposed study by developing a multichannel deep learning architecture with regard to stress detection by leveraging multiple biosignals and also to perform a check upon the interindividuality of the subjects during the learning process.

## 3. Data Preprocessing and Feature Extraction

For the implementation of the multichannel convolutional neural network, multiple prerequisite steps are to be followed. As the data are in the raw format, generalizing the data based on the international system of units remains one of the most primary concerns. Moreover, as the data have been derived from the biosensors, it contains a multitude of abstracted information, which in turn can be difficult for the deep learning algorithms to identify [21]. Therefore, feature engineering on the raw data is to be performed to find the optimum features for the deep learning algorithms to work upon.

### 3.1. Data Set

The data set used in the work was fetched from the UCI machine learning repository that was posted by [10]. The data used in the work were taken from 15 subjects who wore the RespiBAN Professional on the chest and Empatica E4 on the wrist. The RespiBAN was utilized to fetch ECG, EMG, EDA, temperature, accelerometer, and respiration data, whereas Empatica E4 was used to fetch the BVP, EDA, temperature, and accelerometer data. The 15 participants who participated in the data acquisition process were the graduate students of the research facility. The participants chosen for the study were of the mean age of 27.5 ± 2.4 years and out of the 15 participants, 12 were male and 3 were female. For choosing the right candidates, an exclusion principle was introduced where people with pregnancy, chain-smoking, psychological disorders, and cardiovascular disorders were not entertained. For the data acquisition process, the participants were asked to avoid caffeine and tobacco for one hour before the beginning of the procedure. For baseline conditions, the participants were asked to sit or stand near a table and a random magazine was provided to them for reading. For amusement condition, the participants were asked to watch a set of 11 funny video clips where each video clip had a neutral interval of 5 seconds. For determining the stress condition, the participants were exposed to a Trier social stress test (TSST) where the participants were asked to deliver a five-minute speech in front of a panel of three members on personal traits focusing on strength and weaknesses. The participants were provided with a 3-minute interval for the preparation of the speech and were not allowed to refer to their notes while delivering the speech. Post delivering the speech, the participants were asked to count from 2023 to 0 and whenever the participants made a mistake, they were asked to start over. For calibrating the meditation condition, the participants were subjected to a breathing exercise in closed eyes and a comfortable sitting position. And lastly, for the recovery condition, all the sensors were again synchronized using a double-tap gesture and were removed from the participant's body.

### 3.2. Data Conversion and Preprocessing

The data generated from both the wearable devices were in raw format. Therefore, the primary task that had to be performed for getting ahead in the process was to perform the conversion and generalization of the data into the SI units.

#### 3.2.1. Electrocardiography (ECG) Data from the Chest

The ECG data provided by [10] was extracted from the subject by using RespiBAN that was attached to the subjects' chest during the complete experimental procedure. The raw data of the ECG were transformed to its SI unit that is millivolt (mV) using the following formula:

(1) $$\begin{array}{c} \left(\frac{\text{signal}}{\text{Chan\_Bit}}-0.5\right)*V_{\text{CC}}\text{ mV,} \end{array}$$

where the signal is the value at a particular epoch, Chan_Bit refers to the output size, which is equal to 216, and *V*_CC_ refers to the voltage input, which is equal to 3 mV.

#### 3.2.2. Electromyography (EMG) Data from the Chest

The EMG data were extracted from the subject at the sampling rate of 700 Hz from the chest using the RespiBAN device. The raw data of the EMG were converted to its SI unit that is microvolts (*μ*V) using the following formula:

(2) $$\begin{array}{c} \left(\frac{\text{signal}}{\text{Chan\_Bit}}-0.5\right)*V_{\text{CC}}\;µV, \end{array}$$

where the signal is the value at a particular epoch, Chan_Bit refers to the output size which is equal to 216 and *V*_CC_ refers to the voltage input which is equal to 3 *μ*V.

#### 3.2.3. Respiration Data from the Chest

The respiration data were extracted from the subject's chest using the RespiBAN device at 700 Hz of sampling frequency during the experimental procedure. The raw data was converted to a form of displacement percentage using the piezoelectric sensors. The formula for the conversion is as follows:

(3) $$\begin{array}{c} \left(\frac{\text{signal}}{\text{Chan\_Bit}}-0.5\right)*100\%,\; \end{array}$$

where the signal is the value at a particular epoch, and Chan_Bit refers to the output size, which is equal to 216.

#### 3.2.4. Triaxial Accelerometer from the Wrist

The triaxial accelerometer data were captured from the wrist using Empatica E4, which sampled the data to 32 Hz, and the data provided were in the units of 1/64 g. Therefore, the following formula ensures the conversion of the raw data from the triaxial accelerometer to its SI units that is m/s^2^:

(4) $$\begin{array}{c} \frac{\text{signal}*2*9.8}{128}\text{m}/{\text{s}}^{2}. \end{array}$$

#### 3.2.5. Blood Volume Pulse (BVP) from the Wrist

The BVP data are also known as the photoplethysmograph (PPG) data that were extracted from the subjects' wrist using the Empatica E4 at a sampling rate of 64 Hz. The PPG basically narrows down the change in the volume of blood that is being caused by the pressure pulse by illuminating the skin with a light-emitting diode and detecting the amount of light transmitted and reflected back using a photodiode.

#### 3.2.6. Temperature Data from the Wrist

The temperature data from the wrist were pursued using the Empatica E4 device that performed the data generation at a sampling frequency of 4 Hz. The data generated from the subject were in the unit of degree Celsius.

Post conversion and generalization of the raw data to their SI units, the next step that was undertaken was data preprocessing. The data that have been fetched in the study comes from different regions of the subject's body, and multiple devices have been used for the extraction of the data. Moreover, we can observe that there is a lot of variance in terms of the sampling rate of different signals. Therefore, for generalizing the frequencies of all the signals, we tend to convert all the low-sampled signals to 700 Hz initially. Therefore, the triaxial accelerometer data, blood volume pulse, and temperature data have been upsampled to 700 Hz. Now, as the signals have been upsampled to 700 Hz, therefore the data for 15 subjects captured for 100 minutes turned out to be huge in size. So, the signals were further downsampled to 10 Hz by aggregating every 70 samples together using statistical techniques. Also, on the other hand, the labels were downsampled to 10 Hz by taking the mode of the labels for every 70 samples. Finally, after performing all the aggregations and changes in the sampling frequency, the total number of samples of the whole data set for 15 subjects turned out to be 573,480. The distribution of the state of the mind categories has been depicted in Table 1, from which we can find out that the data set is extremely unbalanced in nature.

### 3.3. Feature Engineering

The features that have been engineered from the raw biosignals data are primarily varied in three different forms. The first form is the one-to-one variance or continuous feature variable. In this type of feature, each and every sample of the data set gets an individual value and is continuous in nature. The second form is the subject-wise variance where all the samples of a particular subject are provided with the same value for a particular feature. The third form of feature is based on minute-based variance, where all the samples of a particular minute are provided with the same value. Therefore, using such methods usually provides the features with an optimum variance, which can lead to a better model in terms of generalizability and better classification performance.

The features derived from the ECG, EMG, respiration, and BVP are peak-based features, and the features derived from the accelerometer are purely statistical in nature. The peak-based features for the 1-dimensional biosignals are determined by calculating the local maxima of the cycle of the signal by leveraging the information of the threshold and the definite distance that is needed to be maintained between consecutive peaks.

#### 3.3.1. Electrocardiography (ECG) Features

The features for electrocardiography are basically in the form of minute-based variance where each minute of particular feature gets a different value. Moreover, the features defined in the purpose of ECG are peak-based features as it is a primary notion in terms of biosignals that the peaks of the signal carry a summative value to an entire cycle.

**Figure 1:**
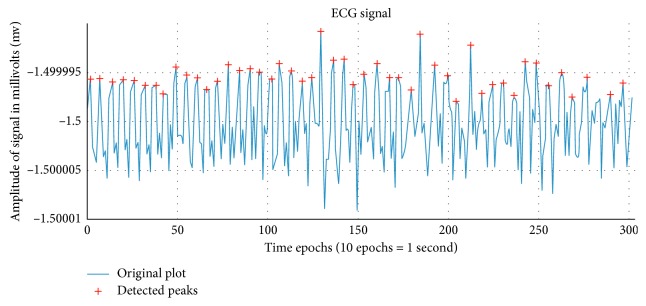
ECG signal for 30 seconds of subject 2.

Figure 1 shows the ECG signal of the second subject for the first 30 seconds, which corresponds to 300 samples as the sampling frequency of the signal was aggregated to 10 Hz. Also, in the figure, we can see the local maximas that have been identified, which further helps to obtain multiple features for the work.

In Table 2, we can observe four features have been mentioned that persuasively points out the patterns in the ECG signal of an individual. Moreover, the features described in the work provides a varied understanding of the subject's nominal peak ranges in the ECG signal, which in turn can be used to determine the state of the subject at a particular instant of time.

#### 3.3.2. Electromyography (EMG) Features

The electromyography signals are well known to measure and record the electrical coefficient of skeletal muscles that tend to define the activation level and figures out the medical abnormalities in a subject. The features calculated for the EMG signal are minute-wise varied to offer an optimum variance across each feature.

**Figure 2:**
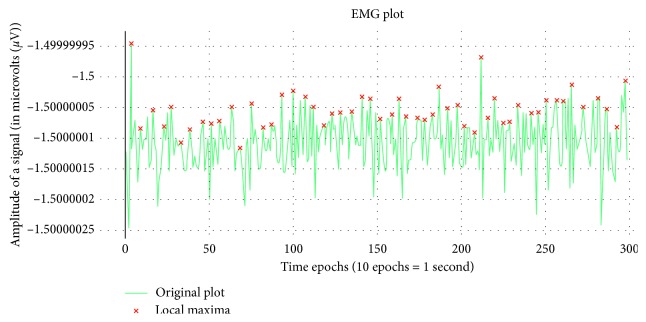
EMG signal for 30 seconds of subject 2.

Figure 2 portrays the EMG signals of subject 2 for the first 30 seconds of the experimental procedure. The red markings shown in the image are the local maxima, which primarily depict the impulsiveness of the electrical coefficient of the skeletal muscles of the subject. Moreover, the peaks or the local maxima tend to identify the pattern in the EMG signals, which can further identify the medical abnormalities in the subject too.

The features demonstrated in Table 3 points out the initial patterns of the EMG signals of a subject, also considering the features based on the peaks of the signals rules in summative information of the signal.

#### 3.3.3. Respiration Features

**Figure 3:**
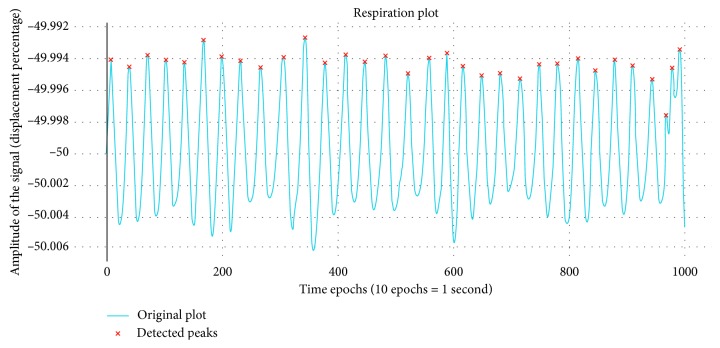
Respiration signal for 100 seconds of subject 2.

The respiration data have been extracted from the chest, which shows the tone and rhythm of the breath and also places the ratio between multiple breath cycles. Also, the respiration data have always been helpful in terms of determining the state of mind and in determining the level of arousal or rate of bio-intensity of a particular subject. The features derived from the respiration data are minute-based such as ECG and EMG.  Figure 3 shows the respiration data for the first 10 seconds during the experimental analysis on subject 2.

Moreover, Table 4 points out the features that depict the patterns across the respiration of the subject. Seeking out the patterns allows for performing some primary anomaly detection on the behavior of the subject across a particular time interval. Therefore, in this work, we considered the usage of respiration signals as a feature to analyze the state of mind of an individual.

#### 3.3.4. Blood Volume Pulse Features

**Figure 4:**
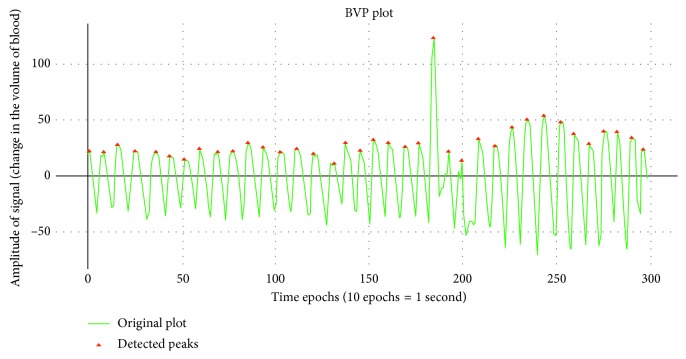
Blood volume pulse for 30 seconds of subject 2.

The BVP signal is specifically derived from the photoplethysmogram that illuminates the skin to determine the changes in the light absorption. From the peaks of BVP, we can determine the heart rate of an individual as every time the heart pumps blood, there is a slight change in the volumetric quantity of blood in arteries, which can be detected using a BVP Signal. In Figure 4, we can see the BVP data that have been plotted for subject 2 for 20 seconds and the peak has been determined.

The features for BVP signal are also varied on the terms of a minute where each minute gets a different value.  Table 5 shows the features that have been used in terms of generating impactful patterns from the BVP data. The trends and the pattern of a particular subject can be readily obtained from the features as it intends to capture the essential details of the signal.

#### 3.3.5. Accelerometer Features

The accelerometer signals are quite reliable in terms of analyzing the level of stress in an individual by seeking out the patterns in the movement [22]. The features are varied with respect to the subjects where all the samples of a particular subject are utilized for the computation of the feature and each subject gets a unique value. The features engineered from the accelerometer signal are being depicted in Table 6 where it can be observed that except correlation-based features, all other features are standalone features. Only the correlation-based are based on the interaction of two axes. Moreover, the features that have been mentioned in Table 6 have been calculated for all the three axes of the accelerometer.

## 4. Deep Learning

### 4.1. Multichannel Convolutional Neural Network Architecture

In recent years, it has been observed how supervised learning techniques have evolved to create some most innovative architectures for solving a particular problem. More evidently, the rise in popularity can be observed for the deep learning algorithms too, which has undergone a major paradigm shift in terms of structure, optimizer functions, and the architecture [23]. Moreover, in the field of health care, the usage of deep learning algorithms has created a reform in terms of image analysis, aneurysm detection in images, biosignals, and a lot more.

In this work, biosignals from chest and wrist wearables have been used for the detection of the state of the mind while undergoing a stress interview. The major significance of this work stands with identifying the stress segment of an individual. For the identification and the predictions of the state of mind, a multichannel convolutional neural network has been used for guaranteeing the optimum generalizability and for identifying complex patterns in the biosignals.

**Figure 5:**
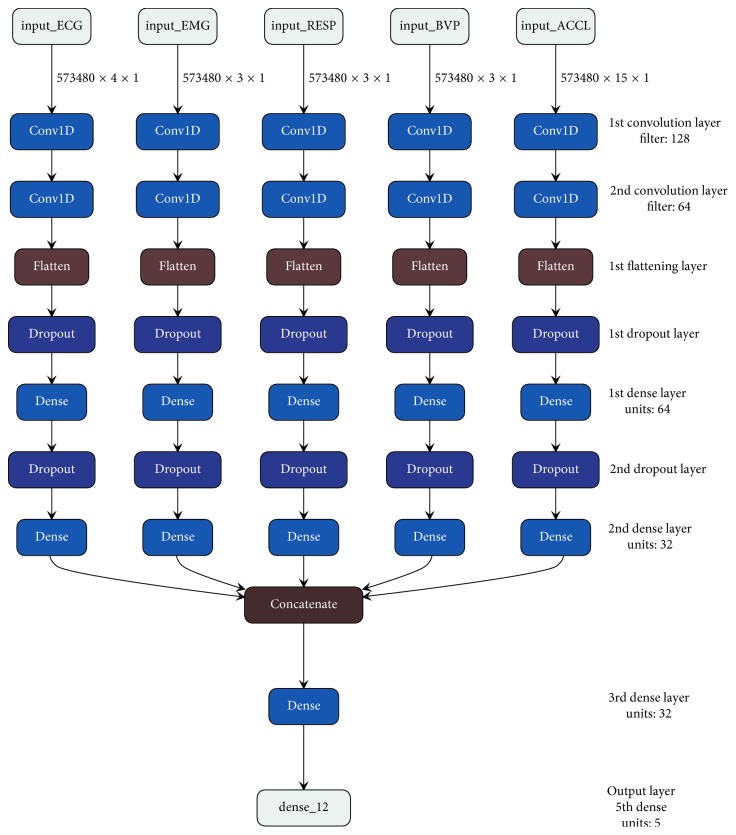
Multichannel CNN architecture.

The model architecture for the multichannel convolutional neural network has been depicted in Figure 5. The architecture shows 5 different input channels for ECG, EMG, respiration, BVP, and accelerometer, respectively. The reason for going forward in separating the channels for different biosignals lies with the fact that the initial feature learning using convolutional neural network for a particular biosignal is being kept discrete with respect to other biosignals for preventing the initial information mixing between individuals [24, 25]. Therefore, the features corresponding to each biosignal such as ECG (4 features), EMG (3 features), respiration (3 features), BVP (3 features), and an accelerometer (15 features) have been coupled respectively and passed along the respective channels.

The most distinctive aspect of convolutional neural networks is the convolution layer, which is used for traversing along the matrix of the data to create a penultimate feature matrix of spatially oriented features using an adaptive kernel or a filter. The adaptive filters for the convolution layers in the multiple channels are to be adjusted on the basis of the input shape of the data matrix. Therefore, the following equation has been used to choose the optimum shape for the filter.

(5) $$\begin{array}{c} f\left(x,1\right)=\left[\text{round}\left(\frac{x}{2}\right)+x\%2\right],\; \end{array}$$

where (*x*, 1) represents the shape of the filter. The equation takes the input size or the number of features used for training as an input, which is denoted by *x*. The term *x* further undergoes a summation of a round function over *x* and a modulo function over *x*, which further provides us the shape of the adaptive filter or the kernel. As in the work we are dealing with 1 − *D* convolutions, the shape of the kernel is always in the form of (*x*, 1).

The feature maps from the first convolution layer are further passed to the second layer of convolution without using any subsampling layer in between. By considering the huge spatial volume of the data that is being trained on the CNN architecture, it can be duly argued that using subsampling layer, such as pooling in between consecutive CNN layers, can make the solution less computationally expensive. But, the usage of subsampling layers for the data whose numerical significance is more important than the spatial arrangement possesses information loss [26]. Therefore, in this architecture, the usage of pooling or subsampling layers has been avoided.

The generated feature matrix by the 2^nd^ convolution layer is then subjected to a fattening layer. The flattening layer first converts the feature matrix from a 2-dimensional matrix to a 1-dimensional array because the subsequent stages of the network contain dense layers. And, for passing a set of data to the dense layer, it is required that the data must be in 1-dimensional format.

After the data are subjected to a flattening layer, they is then subjected to a dropout layer. The dropout layer that has been used in the architecture is basically used for performing regularization and it also assists the model in preventing overfitting. The dropout layer allows the model to fetch for more complex and robust feature relationships by dropping a set of neurons from the visible and the hidden layers to perform more randomized feature learning.

The 6th layer in the architecture is a dense layer, which is the fully connected layer with 64 units. The dense layer allows the model to perform a linear operation on the feature matrix that has been generated by the convolution layer. Moreover, as the convolution layers work locally for the spatial set of defined filters that traverses along with the data matrix, the dense layer acts as a global layer where all the nodes of the layer participate and are connected to all the other nodes in the following layers. Therefore, the usage of dense layers in this work allows the model to establish a global relationship between the features and also accounts for the abstraction of more complex patterns in the data.

The 9^th^ layer in the network is the concatenation layer that allows us to combine the feature matrices from all the channels. The reason behind the concatenation of the feature matrices lies in accordance with our problem statement, which is to detect the state of the mind based upon multiple signals. Therefore, for obtaining the decision threshold based upon all the biosignals, the concatenation of the feature matrices from all the channels is required.

The subsequent layer after the concatenation layer is fully connected layer with 32 units. This fully connected layer is used for fetching out the composite relationships between the concatenated feature matrices from the multiple channels. This layer majorly plots the complex features, complex relationships, and the patterns among the combined feature matrices that support the generation of the decision threshold. The last layer or the output layer that is depicted in Table 7 as well as in Figure 5 consists of 5 units for the 5 classes that are to be predicted namely, baseline condition, amusement condition, stress condition, meditation, and recovery condition. The final dense layer yields the prediction probability of each sample for the 5 classes.

### 4.2. Training Procedure and Cross Validation

The model training in the work used two varied procedures namely Type I and Type II. The type I procedure predominantly was utilized for tuning the hyperparameters and choosing the most viable optimizers for increasing the model performance. Moreover, the type I model was also used to check an initial performance of the model for randomized sequence. For creating the model based on type I procedure, the complete data set was split as 70% of the data were allotted to the training set, 20% were allotted to the validation set and lastly, 10% were allotted to the testing set. The samples that were placed on different sets of data were chosen randomly to remove any correlation in terms of subjects.  Table 8, therefore, points out the number of samples and input features in all the channels for training, validation, and testing in a more constructive way for the type I procedure.

On the other hand, another procedure for training the model was also undertaken by using a cross-validated approach using the data of individual subjects as the testing set. This particular approach was named as Type II procedure. More particularly, for creating the type II model, a 15-fold cross-validation was performed on the data of 15 subjects, where the data of a particular subject were always kept aside for creating the test set. The remaining data of 14 subjects were further allocated to the training and the validation set based on a randomized split with a ratio of 80 : 20. This particular model was developed only for the sake of understanding the capability of the model to generalize across different subjects.  Table 9 demonstrates the number of samples that were used in the training, validation, and testing for each fold by keeping a particular subject's data in the testing set only.

### 4.3. Model Hyperparameters, Loss, and Optimization Functions

The development of a model architecture is one of the prime components of the system that is being developed in the work. But, more advertently, the component that works for the state-of-the-art model architectures is the control over the training process and to optimize the model's performance and outcomes. Therefore, the components such as the model hyperparameters, loss functions, and the optimizer functions are discussed in the following sections.

#### 4.3.1. Model Hyperparameters

The control of the training process is generally held by the hyperparameters that are used for the tuning of the model. As of the current scenario, the optimization of the models by minimizing the testing error is considered to be one of the toughest challenges. But in an intermittent way, the tuning of the elements that reside outside of the model actually influences the complete performance of the model and can be considered as the most challenging part in solving the problem. The primary reason behind the difficulty lies with the fact that the chosen hyperparameters must be model-specific and not training set-specific because hyperparameters that are tuned on the basis of the training set often develop poor model generalizability. Therefore, choosing the right set of hyperparameters is important to maintain the overall tradeoff between model generalizability and optimum objective score.

So, for the choice of right set hyperparameters, Bayesian Sequential Model-Based Optimization (SMBO) is used. Bayesian SMBO is a type of hyperparameter optimization that minimizes a particular objective function by developing a surrogate model (probability function) based on the previous evaluation results of the objective function. The basic objective function of the Bayesian SMBO is given by

(6) $$\begin{array}{c} P\left(\mathrm{score}\;\left|\;\right.\mathrm{hyperparamters}\right)=\frac{P\left(\mathrm{hyperparamters}\left|\;\right.\text{score}\right)P\left(\text{score}\right)}{P\left(\text{hyperparamters}\right)}. \end{array}$$

The surrogate model is considered to be less expensive to be optimized than the main objective function [27]. Therefore, the next set of values that are to be evaluated are selected by using the expected improvement criterion [28]. The expected improvement criterion is defined by:

(7) $$\begin{array}{c} \text{EI}\left(x\right)=E\left(\max\left(f\left(x\right)-f^{*},0\right)\right), \end{array}$$

where *x* belongs to the set of hyperparameter values and considered to be an improvement in the value of the objective function *f*(*x*), and *f*^*∗*^ is the maximum value of the objective that has been observed.

The set of hyperparameters that were obtained by running Bayesian SMBO on the model are

(8) $$\begin{array}{c} \text{learning rate}:\;0.00125,\\ \text{beta}\_1:\;0.9765841,\\ \text{beta}\_2:\;0.8541287,\\ \text{decay}:\;0.000235. \end{array}$$

#### 4.3.2. Model Loss Function

The loss function is a very integral part of the deep learning and the machine learning models. The loss functions are basically used to measure the variability between the predicted output ($\hat{y}$) and the actual value (*y*). The loss functions are nonnegative values that increase the generalizing capability of the model by decreasing the value of the loss function [29]. The basic structure of the loss functions is

(9) $$\begin{array}{c} L\left(\theta \right)\;=\frac{1}{n}\overset{n}{\underset{i=1}{\sum }}L\left(y^{\left(i\right)},f\left(x^{\left(i\right)},\theta \right)\right), \end{array}$$

where *θ* represents the parameters of the model, *x* represents the feature matrix of the model, and *y* represent the actual labels of the model.

The loss function used in the work is the categorical cross-entropy loss, which is also known as the SoftMax loss. In the categorical cross-entropy loss function, each prediction is compared to the actual class value and a score is calculated. The score is further used to penalize the probability of the prediction based on the difference from the actual value. The penalty that is offered to the predicted value is purely logarithmic in nature where a small score is allotted to tiny differences and the huge score is allotted to larger differences [30]. The equation for the categorical cross-entropy loss is given by

(10) $$\begin{array}{c} L\left(y,\hat{y}\right)\;=-\frac{1}{n}\overset{n}{\underset{i=1}{\sum }}\overset{c}{\underset{j=1}{\sum }}\left(y_{i,j}\log\left({\hat{y}}_{i,j}\right)\right), \end{array}$$

where the double sum has been performed on the ith data samples ranging from 1 to *N* and the classes that range from 1 to *C*. The term *y*_*i*,*j*_ in the equation corresponds to the actual one hot encoded label at *i*^th^ index of *j*^th^ category. And the term ${\hat{y}}_{i,j}$ corresponds to the prediction of the model for the samples as *i*^th^ index [30].

#### 4.3.3. Model Optimizer Functions

The optimizer functions are the ones that play an integral part in the optimization of the internal parameters of a model. The internal parameters of the type of model that is being dealt with in the work are the weights and biases. Now, in the previous segment, we have discussed the loss function of the model that needs to be minimized over the training iterations. But the loss function is more of a mathematical way of determining what is the error rate between the predictions and the actual labels. Therefore, optimizer functions are used to incorporate the loss function with the models' internal parameters such as weight and biases for updating the same based on the response generated from the loss functions.

In this work, multiple optimizer algorithms were used and a comparative analysis was performed with regard to which optimizer function relates to the best minimization of the categorical cross-entropy loss and ties best with the hypothesis of the problem. The comparative analysis can be seen in Table 10 between the multiple optimizer functions and the best optimizer for the problem statement was found to be Adam optimizer.

## 5. Results

The multichannel convolutional neural network model developed in the work aimed to provide very sound and effective results on the basis of the classification of the different state of minds for a particular subject. Also, the model developed in the work provided with the results by prompting an average recall and precision of 97.238% and 97.652%, respectively, for all the classes. The model also showcased a constant tendency of precision and recall in the random data folds of training and testing.

Moreover, with prior accordance to the hypothesis that was developed in the initial phase stated the rules that the precision and recall of all the class must be above the same threshold providing a fixed classification rate in all classes. As in the previous work [10] we have seen that the hypothesis tends to prioritize more on the third class, which is the stress, but in this work, we tend to predict the samples of all the five classes precisely.

**Figure 6:**
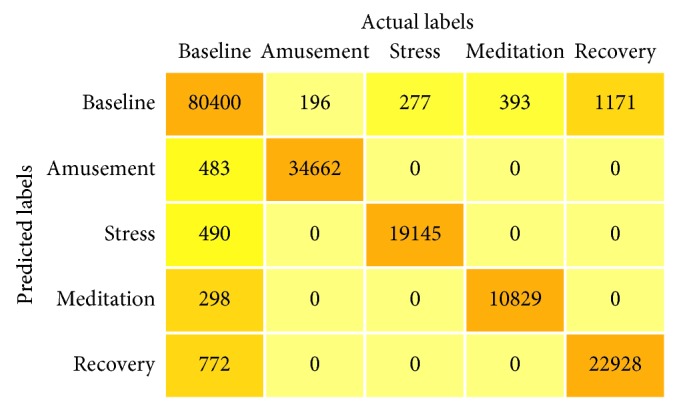
Confusion matrix of the multichannel CNN model.

In Figure 6, we can see the confusion matrix that has been derived on the basis of the classification results of the test data set. Therefore, we can see from the confusion matrix that it has full accordance with the hypothesis with regard to the correct true positives and true negatives of each class. But in Figure 6, it can be observed that the baseline class has a greater number of mispredictions than other classes and also other classes have got a lot of the samples that have been mispredicted to the baseline class. The primary reason behind such an incident is that the features of the data sample belonging to the baseline class have a strong correlation with features of the data samples belonging to other classes. But, such a scenario can be avoided by lowering the prediction threshold from 0.2 of all the classes except the baseline class, which in turn will reduce the mispredictions in the baseline class. However, dampening of the prediction threshold of the classes may lead to an invariant scenario of less generalizability of the model. Therefore, to maintain a tradeoff between the correct predictions and the mispredictions, the situation is been kept as it is.

The metrics used for evaluating the potential of the model are precision, recall, and the F1 score of all the classes. In the current scope of this work, the recall of each class provides us the information, with regard to the number of data samples that the model has correctly predicted to be of a particular class. The precision on the other hand of a particular class determines the confidence of prediction to belong to a particular class. And lastly, the F1 score suggests the weighted average of both precision and recall and therefore takes a leap over all the wrongly predicted samples of a particular class.

**Figure 7:**
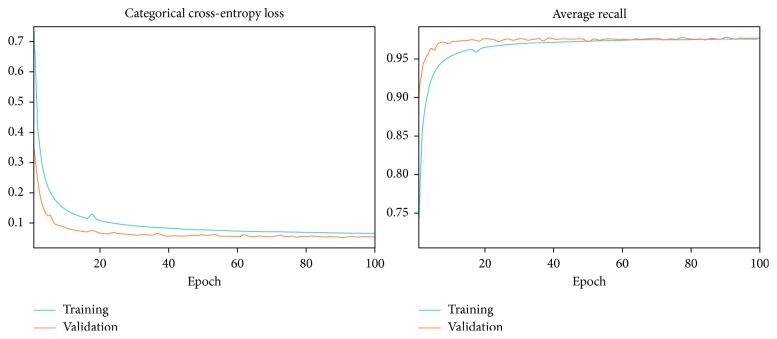
Model training process using Adam optimizer for 100 epochs.

Figure 7 shows the training and validation convergence of the model. The model iteration that has been shown in the figure is the final model that is trained with the hyperparameters mentioned in Section 4.3.1 and the optimizer algorithm Adam.

Table 10 puts forward the classification report of the model with respect to different optimizer algorithms that were used to optimize the internal parameters of the model. From the table, it can be seen that all the three optimization algorithms namely, Adam, RMSprop, and SGD provided us with decent results. But, for the creation of the final binary serialized object of the model, Adam was selected. The reason for choosing Adam in this scenario lies behind a few reasons:

- The overall performance of the Adam Optimized model is better than the other two.
- The model optimization is very much time-efficient.
- The model optimization is computationally efficient.
- As the type of data, we are dealing within the work, there is no prospect for an upper bound or lower bound of a particular type of biosignal. Therefore, for reproducibility of the model in the future, it may happen that the gradients might change for a particular type of subject. So, having an algorithm to optimize the model which is not varied by the rescaling of the gradient will turn out to be useful [31].

Table 11 plots the comparative analysis between the performance of the multichannel convolutional neural network and conventional single-channel convolutional neural network. Both the networks have been trained with the same optimizer function that is Adam but for the single-channel convolutional neural network, a different set of hyperparameters were used, which were derived by using the same Bayesian SMBO. From Table 11, it can be evidently observed that the single-channel also performed respectively well. But, the performance of the multichannel convolutional neural outperformed that of the single-channel convolutional network. Moreover, in the “meditation class” of the single-channel convolutional neural network, it can be observed that the recall is pretty low than other classes. The lower value of the recall for the meditation class is because there are comparatively a smaller number of samples in the meditation class than other classes. Therefore, it was found that the multichannel convolutional neural network overcomes the hurdle regarding such imbalanced classification where there is an identifiable disparity in the number of samples across the classes.

Table 12 depicted below further shows the model performance of the multichannel convolutional neural network model, which was trained using the cross-validated approach or type II model. The type II model seemed to provide decent results. But, we can see that there is quite a difference between the performance level depicted in Table 11 by the Type I model where the training, validation, and testing sets were randomized samples and in Table 12 by the Type II model where the sample of a particular subject is only on the testing set. The primary reason behind this deviation in the model performance is that every subject has altogether different kind of thresholds when it comes to biosignal-based predictions.

## 6. Discussion

In the present world, as the life of people have changed in a varied way where they are much suited to the new customized lifestyle and the disorientation of the biological clock, it has been very necessary and of paramount importance that the state of mind and health must be maintained properly. But, people these days have turned out to be more reluctant to spend their time with the therapists or the doctors for pursuing a proper check on their health. Therefore, with the emergence of smart healthcare, the process could be very much maintained and measured using the wearable devices that have grown into much affluence in society. We know that the smart wearables that have presently arrived in the sector support multiple biosignals of the user such as movement, heart rate variability, pulse pressure, vascular respiration, perfusion index, etc. Therefore, these biosignals, if properly monitored for a particular subject, will be able to identify the health conditions as well as will be able to detect the primary anomalies in the health.

The data that have been used in the work have been properly curated from the wearable device worn by the subject during the experimental process for detecting the certain state of mind that can be very much useful to understand the mental conditions of the subject. In the data amalgamation process, five key classes were noted namely, recovery, baseline, stress, amusement, and meditation. And for the classification purpose, multiple biosignals were utilized such as accelerometer, electrocardiography, electromyography, blood volume pulse, and body temperature. The signals were further analyzed to perform optimum feature engineering where the summative information of complete signals is extracted using the maxima and the minima of the signal at a particular instance of time.

For the classification purpose, a multichannel convolutional neural network architecture was developed in the work. The primary concern for the development of a multichannel architecture is that as we have different biosignals from different parts of the body, we tried to avoid the initial intermixing of the features of different biosignals. But later on, at the penultimate region, the feature matrixes conceived by different channels are concatenated for pursuing an integrated decision threshold for the detection of the state of the mind from all the biosignals. But at a certain point, a question can be raised that “Why deep learning has been used for solving the particular problem?” The answer to the question lies in the fact that as the biosignals are of an abstract nature and there are multiple complex interactions and patterns in the data, manually engineering the right features would be very difficult. Therefore, in this work, deep learning is performed as the method has the ability to produce extremely complex feature representations and also allows model reproducibility, which will allow us to perform incremental learning if a certain new set of data arrives.

## 7. Conclusion

In the proposed study, a multichannel convolutional neural network architecture was developed for the detection of state of the mind by leveraging biosignals from the wearable devices. The different types of biosignals used in the work are electrocardiography, electromyography, respiration, blood volume pulse, and accelerometer. The model developed performed pretty well by prompting an average recall and precision of 97.238% and 97.652%, respectively, across all the classes. In the work, a comparative analysis was performed for choosing the right optimizer by keeping in mind the performance of the optimizer with respect to the cost of computation, time efficiency, and model reproducibility. Finally, it was found that the model optimized with Adam optimizer performed the best with respect to the other optimizer functions.

To conclude, the outcome of the study is very motivating. However, in the area of classification of the state of the mind and the analysis of the biosignals, there is still a huge scope for further research. Therefore, it is very much recommended to investigate multiple ways of solving the particular type of problem and to understand the complete capability of multichannel deep learning architectures, which will further impact the society in a novel and a positive way.

## Figures and Tables

**Table 1:** State of the mind category distribution.

State of the mind class	Number of samples
Baseline	274,790
Amusement	117150
Stressed	65450
Meditation	37090
Recovery	79000

**Table 2:** Electrocardiography (ECG) features.

Feature name	Description
ECG_Peaks	This gives out the number of local maxima in a minute
ECG_Average_Amplitude	This feature gives out the average amplitude of the local maximas in a minute
ECG_Differ_Mean	This feature pursues the average difference between consecutive local maxima in a minute
ECG_Resting	This feature shows out the resting motion of a subject, which means the number of local maxima within 10 samples that is 1 second

**Table 3:** Electromyography (EMG) features.

Feature name	Description
EMG_Peaks	This gives out the number of local maxima in a minute
EMG_Average_Amplitude	This feature gives out the average amplitude of the local maximas in a minute
EMG_Differ_Mean	This feature pursues the average difference between consecutive local maxima in a minute

**Table 4:** Respiration features.

Feature name	Description
RESP_Peaks	Number of breath cycles in a minute
RESP_Average_Amplitude	This feature gives out the average amplitude of the local maximas in a minute
RESP_Differ_Mean	This feature pursues the average difference between consecutive local maxima in a minute

**Table 5:** Blood volume pulse features.

Feature name	Description
BVP_Peaks	This gives out the number of local maxima in a minute
BVP_Average_Amplitude	This feature gives out the average amplitude of the local maximas in a minute
BVP_Differ_Mean	This feature pursues the average difference between consecutive local maxima in a minute

**Table 6:** Accelerometer signal features.

Feature	Equation	Description
Mean	$\bar{x}=\left(1/n\right)\sum_{i=0}^{n}\left(x_{i}\right)$	The mean of the signal for each subject
Standard deviation	$\sigma =\sqrt{1/N\sum_{i=1}^{N}{\left(x_{i}-\bar{x}\right)}^{2}}$	The standard deviation of the signal is calculated for each value
Correlation	$\text{Corr }=\left(1/\left(N-1\right)\right)\sum_{n=1}^{N}\left(\left(x_{i}-\bar{x}\right)\left(y_{i}-\bar{y}\right)\right)/\left(\text{std}\left(x\right)\mathrm{*std}\left(y\right)\right)$	The correlation coefficient between the two accelerometer signals
Kurtosis	$\text{Kurt}\left(x\right)=\left(E\left[{\left(x-\bar{x}\right)}^{4}\right]/\text{std}{\left(x\right)}^{2}\right)-3$	Kurtosis shows the peakedness of a signal
Crest factor	$\text{Crest}\left(x\right)=\left(\left(\text{max}\left(x\left(n\right)\right)\right)/\left(\sqrt{\left(1/\left(N-1\right)\right)\sum_{n=1}^{N}a{\left(n\right)}^{2}}\right)\right)$	It shows the signal impulsiveness with the maximum accelerometer value

**Table 7:** Multichannel CNN architecture.

Layer	Layer type	Filters	Size	No. of parameters	Output dimension	Activation
1	Input	—	—	—	ECG: (4, 1) EMG: (3, 1) RESP: (3, 1) BVP: (3, 1) ACCL: (15, 1)	—

2	Conv1D (1st layer)	128	ECG: (2, 1) EMG: (2, 1) RESP: (2, 1) BVP: (2, 1) ACCL: (8, 1)	ECG: 384 EMG: 384 RESP: 384 BVP: 384 ACCL: 1152	ECG: (3, 128) EMG: (2, 128) RESP: (2, 128) BVP: (2, 128) ACCL: (8, 128)	ReLU

3	Conv1D (2nd layer)	64	ECG: (2, 1) EMG: (2, 1) RESP: (2, 1) BVP: (2, 1) ACCL: (8, 1)	ECG: 16448 EMG: 16448 RESP: 16448 BVP: 16448 ACCL: 65600	ECG: (2, 64) EMG: (1, 64) RESP: (1, 64) BVP: (1, 64) ACCL: (1, 64)	ReLU

4	Flatten		—	—	ECG: 128 EMG: 64 RESP: 64 BVP: 64 ACCL: 64	—

5	Dropout	—	—	—	—	

6	Dense (1st layer)	64	—	ECG: 8256 EMG: 4160 RESP: 4160 BVP: 4160 ACCL: 4160	ECG: 64 EMG: 64 RESP: 64 BVP: 64 ACCL: 64	ReLU

7	Dropout	—	—	—	—	—

8	Dense (2nd layer)	32		ECG: 2080 EMG: 2080 RESP: 2080 BVP: 2080 ACCL: 2080	ECG: 32 EMG: 32 RESP: 32 BVP: 32 ACCL: 32	ReLU

9	Concatenate	—		0	160	

10	Dense (3rd layer)	32	160	5152	32	ReLU

11	Dense (output)	5	32	165	5	SoftMax

**Table 8:** Training, validation, and testing divisions for all the channels and number of features for Type I.

Channel	Training samples	Testing samples	Validation set	No. of features
ECG channel	616,413	176,118	88,059	4
EMG channel	616,413	176,118	88,059	3
Respiration channel	616,413	176,118	88,059	3
BVP channel	616,413	176,118	88,059	3
Accelerometer channel	616,413	176,118	88,059	15

**Table 9:** Number of samples for each fold of training.

The subject in the test set	Training set	Validation set	Testing set
Subject 1	656,872	164,218	59,500
Subject 2	654,184	163,546	62,860
Subject 3	654,264	163,566	62,760
Subject 4	655,896	163,974	60,720
Subject 5	649,320	162,330	68,940
Subject 6	663,744	165,936	50,910
Subject 7	661,960	165,490	53,140
Subject 8	664,144	166,036	50,410
Subject 9	661,728	165,432	53,430
Subject 10	663,824	165,956	50,810
Subject 11	650,536	162,634	67,420
Subject 12	658,360	164,590	57,640
Subject 13	654,512	163,628	62,450
Subject 14	653,984	163,496	63,110
Subject 15	659,280	164,820	56,490

**Table 10:** Comparative analysis of the model performance based on the optimizer algorithms for subject 1 in the testing set.

Metric	Adam	RMSprop	SGD
Accuracy	97.62	90.45	92.51
Recall “baseline”	0.9861	0.8945	0.9063
Precision “baseline”	0.9703	0.9106	0.9542
F1 score “baseline”	0.9716	0.9033	0.9311
Recall “amusement”	0.9891	0.9322	0.9256
Precision “amusement”	0.9956	0.9158	0.9428
F1 score “amusement”	0.991	0.9288	0.9299
Recall “stress”	0.9832	0.9647	0.9568
Precision “stress”	0.9784	0.94	0.9487
F1 score “stress”	0.9693	0.9561	0.9509
Recall “meditation”	0.9583	0.9428	0.9467
Precision “meditation”	0.9752	0.9022	0.9788
F1 score “meditation”	0.9680	0.9312	0.9635
Recall “recovery”	0.9456	0.9365	0.9387
Precision “recovery”	0.9711	0.9174	0.9579
F1 score “recovery”	0.9620	0.9258	0.9466

**Table 11:** Comparative analysis of the model performance for multichannel CNN and single-channel CNN for subject 1 in the testing set.

Metric	Multi channel CNN	Single channel CNN
Accuracy	97.62	87.53
Recall “baseline”	0.9861	0.9524
Precision “baseline”	0.9703	0.9347
F1 score “baseline”	0.9716	0.9435
Recall “amusement”	0.9891	0.9311
Precision “amusement”	0.9956	0.9006
F1 score “amusement”	0.991	0.9132
Recall “stress”	0.9832	0.8991
Precision “stress”	0.9784	0.9157
F1 score “stress”	0.9693	0.9036
Recall “meditation”	0.9583	0.7658
Precision “meditation”	0.9752	0.8631
F1 score “meditation”	0.9680	0.8122
Recall “recovery”	0.9456	0.9136
Precision “recovery”	0.9711	0.9217
F1 score “recovery”	0.9620	0.9178

**Table 12:** Comparative analysis of the model performance for multichannel CNN for Type-II model.

Metrics	Subject 1	Subject 2	Subject 3	Subject 4	Subject 5	Subject 6	Subject 7	Subject 8	Subject 9	Subject 10	Subject 11	Subject 12	Subject 13	Subject 14	Subject 15
Accuracy	75.34	78.66	74.56	0.7644	75.84	76.54	77.8	79.89	78.26	76.20	76.79	77.72	77.66	78.08	76.14
Recall “baseline”	0.724	0.79	0.741	0.748	0.739	0.873	0.793	0.874	0.814	0.813	0.791	0.831	0.884	0.83	0.719
Precision “baseline”	0.752	0.795	0.771	0.715	0.769	0.718	0.798	0.715	0.79	0.727	0.742	0.809	0.8	0.775	0.713
F1 score “baseline”	0.738	0.792	0.756	0.731	0.754	0.788	0.795	0.787	0.802	0.768	0.766	0.82	0.84	0.802	0.716
Recall “amusement”	0.723	0.75	0.718	0.676	0.669	0.694	0.678	0.705	0.709	0.694	0.676	0.686	0.714	0.682	0.728
Precision “amusement”	0.727	0.813	0.818	0.841	0.81	0.835	0.714	0.769	0.752	0.742	0.777	0.816	0.844	0.79	0.736
F1 score “amusement”	0.725	0.78	0.765	0.75	0.733	0.758	0.696	0.736	0.73	0.717	0.723	0.745	0.774	0.732	0.732
Recall “stress”	0.719	0.734	0.786	0.74	0.804	0.718	0.744	0.744	0.806	0.734	0.767	0.749	0.746	0.747	0.801
Precision “stress”	0.785	0.796	0.769	0.788	0.843	0.781	0.836	0.778	0.813	0.785	0.815	0.844	0.805	0.76	0.838
F1 score “stress”	0.751	0.764	0.777	0.763	0.823	0.748	0.787	0.761	0.809	0.759	0.79	0.794	0.774	0.753	0.819
Recall “meditation”	0.734	0.798	0.775	0.873	0.779	0.756	0.835	0.849	0.759	0.788	0.757	0.819	0.766	0.791	0.773
Precision “meditation”	0.846	0.808	0.822	0.811	0.843	0.822	0.851	0.776	0.778	0.841	0.785	0.834	0.862	0.871	0.887
F1 score “meditation”	0.786	0.803	0.798	0.841	0.81	0.788	0.843	0.811	0.768	0.814	0.771	0.826	0.811	0.829	0.826
Recall “recovery”	0.867	0.861	0.723	0.785	0.801	0.786	0.84	0.819	0.825	0.781	0.849	0.801	0.773	0.854	0.786
Precision “recovery”	0.814	0.841	0.803	0.795	0.837	0.782	0.808	0.836	0.81	0.798	0.795	0.814	0.789	0.785	0.824
F1 score “recovery”	0.84	0.851	0.761	0.79	0.819	0.784	0.824	0.827	0.817	0.789	0.821	0.807	0.781	0.818	0.805

## Data Availability

The data used to support the experiments and the findings of the study have been duly included in Section 3.1.
